# Comment on Watson et al. Alliance for Sleep Clinical Practice Guideline on Switching or Deprescribing Hypnotic Medications for Insomnia. *J. Clin. Med*. 2023, *12*, 2493

**DOI:** 10.3390/jcm14051634

**Published:** 2025-02-28

**Authors:** Margaret Moline, Jocelyn Y. Cheng, Jane Yardley, Kate Pinner

**Affiliations:** 1 Eisai Inc., 200 Metro Blvd., Nutley, NJ 07110, USA; jocelyn_cheng@eisai.com; 2 Eisai Ltd., Hatfield AL10 9SN, UK; jane_yardley@eisai.net (J.Y.); kate_pinner@eisai.net (K.P.)

Health authorities and medical communities in many countries are discouraging the use of benzodiazepines and non-benzodiazepine hypnotics for patients with insomnia. Therefore, the publication of the review article by Watson et al. [1] was a welcome addition to the literature.

However, there is a misinterpretation of results from a clinical study of lemborexant (E2006-G000-303, NCT02952820, SUNRISE 2 [2]) that appears in Table 1 of the aforementioned manuscript. The table states that after discontinuing treatment with lemborexant, participants were “[s]ignificantly worse than end of double-blind treatment for two weeks after discontinuation for SOL, but for WASO, [were] only significantly worse than end of double-blind treatment for the first week”.

While the source for the table is correctly cited as Takaesu et al. [3], the claim of Watson and colleagues is not supported by the data for several fundamental reasons. First, as an acceptable alternative to a statistical analysis, changes in values for the sleep parameters were assessed by determining whether the 95% confidence intervals for the least squares mean value were overlapping between each timepoint. This approach was needed due to the absence of a placebo comparator, and therefore no statistical comparison between each timepoint was performed in the analysis by Takaesu et al., as is incorrectly stated by Watson et al.

Second, Watson et al. conflate the findings from one timepoint (second week off treatment) in one group of participants (lemborexant 5 mg for 12 months continuously [LEM5-LEM5]) with results for all participants off treatment. According to the 95% confidence interval assessment, as can be seen in Figure 2 from Takaesu et al. [3] (see Figure 1), subjective sleep onset latency (sSOL) was not different at the end of treatment during either week of follow-up in participants taking lemborexant 10 mg (LEM10-LEM10).

For participants taking LEM5-LEM5, sSOL was only remarkably different during the second week off treatment compared with the end of the 12-month double-blind treatment period. Since our paper did not combine data from LEM5-LEM5 and LEM10-LEM10 participants, it is unclear how the authors arrived at their conclusion.

Furthermore, at no time during the follow-up period did the subjective wake after sleep onset (sWASO) values differ from the end of treatment. This can also be seen in Figure 2 from Takaesu et al. (see Figure 1), as the confidence intervals clearly overlap during each of the two follow-up weeks compared with sWASO at the end of double-blind treatment. It should also be noted that health authorities agree there is no evidence of rebound associated with the use of lemborexant [4].

Given these clarifications, the description of effects following abrupt discontinuation of lemborexant should be similar to that of suvorexant and daridorexant. A corrigendum to correct these misinterpretations seems warranted.

## Figures and Tables

**Figure 1:**
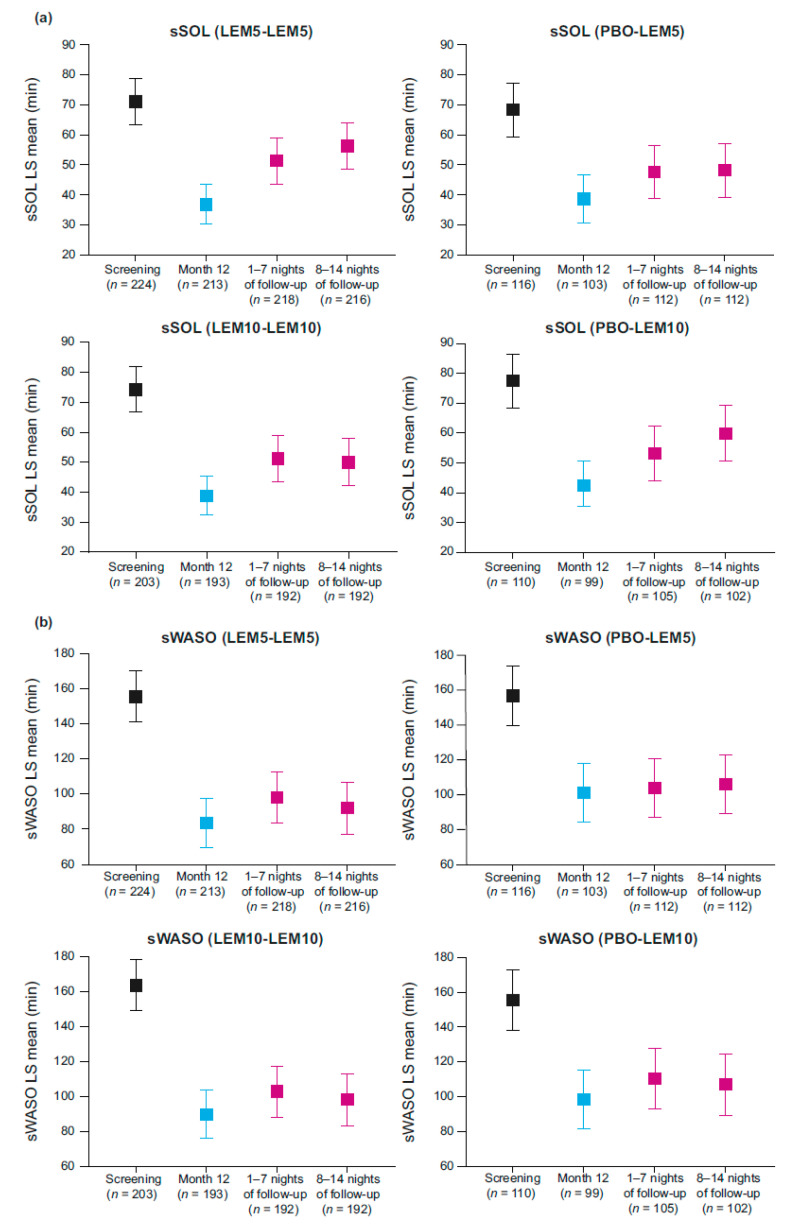
Sleep outcomes from Study E2006-G000-303 (Study 303) at screening, month 12, and during the 2-week follow-up period [3]. Comparison of 95% CI for (**a**) sSOL and (**b**) sWASO. Month 12 and during follow-up in subjects who completed 6 or 12 months of LEM5 or LEM10 (6- and 12-month active completers analysis sets). Error bars represent 95% CI. CI, confidence interval; LEM5, lemborexant 5 mg; LEM10, lemborexant 10 mg; LS, least squares; PBO, placebo; sSOL, subjective sleep onset latency; sWASO, subjective wake after sleep onset. Reprinted with permission from Takaesu et al., 2023 [3].

## References

[1] Watson N.F., Benca R.M., Krystal A.D., McCall W.V., Neubauer D.N. (2023). Alliance for Sleep Clinical Practice guideline on switching or deprescribing hypnotic medications for insomnia. J. Clin. Med..

[2] Kärppä M., Yardley J., Pinner K., Filippov G., Zammit G., Moline M., Perdomo C., Inoue Y., Ishikawa K., Kubota N. (2020). Long-term efficacy and tolerability of lemborexant compared with placebo in adults with insomnia disorder: Results from the phase 3 randomized clinical trial SUNRISE 2. Sleep.

[3] Takaesu Y., Suzuki M., Moline M., Pinner K., Inabe K., Nishi Y., Kuriyama K. (2023). Effect of discontinuation of lemborexant following long-term treatment of insomnia disorder: Secondary analysis of a randomized clinical trial. Clin. Transl. Sci..

[4] Dayvigo Full Prescribing Information. https://www.dayvigo.com/-/media/Files/DAYVIGO/PDF/prescribing-information.pdf?hash=b773622b-b11d-419e-9dee-068c55a90aab.

